# Alteration of Gut Microbiota Relates to Metabolic Disorders in Primary Aldosteronism Patients

**DOI:** 10.3389/fendo.2021.667951

**Published:** 2021-08-17

**Authors:** Yu Liu, Qingyao Jiang, Zhihong Liu, Sikui Shen, Jianzhong Ai, Yuchun Zhu, Liang Zhou

**Affiliations:** Department of Urology, Institute of Urology (Laboratory of Reconstructive Urology), West China Hospital, Sichuan University, Chengdu, China

**Keywords:** primary aldosteronism, gut microbiota, metabolic disorders, diabetes mellitus, obesity

## Abstract

**Purpose:**

This study aimed to determine the relationships among gut microbiota, primary aldosteronism (PA), and related metabolic disorders.

**Methods:**

The study enrolled 13 PA patients, 26 sex-matched primary hypertension patients, and 26 sex-matched healthy controls. Demographic and clinical characteristics such as age, body mass index (BMI), blood aldosterone–renin ratio, blood potassium, blood glucose, blood lipid parameters, and history of diabetes mellitus (DM) were compared between the three groups. The gut microbiota of each participant was examined by 16S rRNA gene sequencing. Spearman correlation analysis was performed to demonstrate the relationship between gut microbiota and clinical characteristics.

**Results:**

BMI and the percentage of DM in PA patients were higher than those in healthy controls (*p* < 0.05), but not higher than those in primary hypertension patients (*p* > 0.05). The gut microbiota of healthy controls and primary hypertension patients had a higher alpha diversity level than that of PA patients. PA patients had fewer short-chain fatty acid (SCFA)-producing genera (*Prevotella*, *Blautia*, *Coprococcus*, *Anaerostipes*, and *Ruminococcus*) and more inflammation-associated genera (*Megamonas*, *Sutterella*, and *Streptococcus*) than healthy controls (*p* < 0.05). The gut microbiota of PA patients was more inclined to encode microbial pathways involved in sugar metabolism, such as starch and sucrose metabolism and fructose and mannose metabolism. Blood potassium was negatively correlated with the relative abundance of *Romboutsia* (*R* = −0.364, *q* = 0.023). Diastolic blood pressure (DBP) was positively correlated with *Romboutsia* (*R* = 0.386, *q* = 0.015). Systolic blood pressure (SBP) was negatively correlated with *Blautia* (*R* = −0.349, *q* = 0.030).

**Conclusions:**

The alteration of gut microbiota in PA patients, especially bacteria and pathways involved in inflammation, SCFAs, and sugar metabolism, may be associated with chronic metabolic disorders.

## Introduction

Primary aldosteronism (PA) is the most common cause of secondary hypertension (1). PA is frequently poorly diagnosed and treated, leading to aldosterone-specific morbidity and mortality. Many observational studies have reported an increased prevalence of metabolic complications [such as diabetes mellitus (DM) and obesity] among PA patients (2, 3). Hyperaldosteronemia, hypokalemia, and cortisol secretion are regarded as risk factors for metabolic disorders (4). However, a clear pathophysiological interrelationship linking the two entities has yet to be established.

The gut microbiome is part of a complex ecosystem. Many studies have shown that the gut microbiota and its metabolites are involved in some metabolic diseases, such as metabolic syndrome, DM, and obesity (5–8). Since the reasons for high prevalence of metabolic diseases in PA patients have not been well described, it will be interesting to explore the interaction between PA-induced metabolic disorders and independent microbial characteristics. To address this problem, we conducted a case–control study with 65 participants in which we analyzed gut microbiota to establish the intestinal microbial profiles of PA patients. We aimed to determine the relationship between gut microbiota, PA, and related metabolic diseases.

## Materials and Methods

### Study Population

A case–control study was performed at West China Hospital, Sichuan University in China from September 2019 to December 2019. We recruited PA patients, primary hypertension patients, and healthy controls. PA patients (PA group) were primarily diagnosed by the aldosterone–renin ratio (cutoff value = 30), and then confirmed by an intravenous saline load test (cutoff value of postsaline aldosterone = 5 ng/dl) and a captopril challenge test (cutoff value of postcaptopril aldosterone = 11 ng/dl). Abdominal computed tomography was performed to determine the location of the adrenal nodules. Adrenal venous sampling was performed to preoperatively distinguish unilateral and bilateral PA. Primary hypertension patients (hypertension group) were defined as patients with hypertension who had systolic blood pressure (SBP) ≥140 mmHg or diastolic blood pressure (DBP) ≥90 mmHg. Blood cortisol, catecholamine and renin–angiotensin–aldosterone, thyroid hormone, thyroid-stimulating hormone, and parathyroid hormone were examined to verify that they did not have endocrine hypertension. Acromegaly and obstructive sleep apnea were also considered if patients reported related symptoms (1). Healthy controls (control group) were defined as participants without hypertension, with SBP  ≤139 mmHg and DBP ≤89 mmHg. Blood pressure was measured in a sitting position by nurses or physicians. Three readings were recorded at 5-min intervals with a random-zero mercury column sphygmomanometer, and the ;average was taken as the final measurement. All PA patients received laparoscopic adrenalectomy, with the pathological specimen confirmed as cortical adenoma. This study was approved by the West China Hospital, Sichuan University Medical Research Ethics Committee (IRB approval number 2018182), and informed consent was obtained from each participant.

Participants were excluded if they had the following conditions: the use of antibiotics within 3 months before fecal sampling, inflammatory bowel disease, irritable bowel syndrome, digestive tract infection, tumors of the digestive system, intestinal surgery, and recurrent diarrhea or constipation within 1 month before stool sample collection.

### Collection of Demographic and Clinical Characteristics

A questionnaire was designed to query the demographic and clinical information of participants, including sex, age, and history of smoking and drinking. Weight and height were measured by instruments. Body mass index (BMI) was calculated by dividing weight in kilograms by the square of height in meters. Blood pressure, blood aldosterone–renin ratio, blood potassium, blood glucose, blood lipid parameters [triglyceride, cholesterol, high-density lipoprotein (HDL) and low-density lipoprotein (LDL)], and history of DM were obtained from medical records. Fecal samples of the study participants were collected at inpatient or outpatient departments when PA and primary hypertension patients visited our hospital. We asked healthy controls from the health examination center of our hospital to provide stool samples voluntarily. The samples were immediately frozen in liquid nitrogen and subsequently stored at −80°C until analysis.

### Analyses of Gut Microbiota

Microbial DNA was extracted from fecal samples using a QIAamp Fast DNA Stool Mini Kit. Negative controls were conducted using sterile water to exclude any possible contamination. The V3–V4 regions of the 16S rRNA gene were amplified with primers 338 F (5′-ACTCCTACGGGAGGCAGCAG-3′) and 806R (5′-GGACTACHVGGGTWTCTAAT-3′). We constructed a library using the NEXTFLEX Rapid DNA-Seq Kit. Amplicons were pooled in equimolar amounts and paired-end sequenced (2 × 300) on an Illumina MiSeq platform. After demultiplexing, the resulting sequences were merged by FLASH (version 1.2.11) (9) and quality filtered with fastp (version 0.19.6) (10). Then the sequences were denoised using the DADA2 (11) plugin in QIIME2 (version 2020.2) (12). The denoised sequences are called amplicon sequence variants (ASVs). Taxonomic assignment of ASVs was performed with the SILVA 16S rRNA gene database (version 138).

Based on the sequencing data, the coverage index was first calculated to determine if the sequencing depth covered the whole bacterial diversity. The higher the coverage index is, the higher the probability of the sequence being detected. We compared the alpha diversity indices, such as the Shannon and Simpson indices between the control, hypertension, and PA groups. A higher Shannon index and a lower Simpson index indicate a higher richness and evenness of the gut microbiota. Interindividual variability (beta diversity) among these three groups was also evaluated with principal coordinates analysis (PCoA) by Bray–Curtis distance, the permutational multivariate analysis of variance (PERMANOVA) test, and the analysis of similarities (ANOSIM) test. The linear discriminant analysis (LDA) effect size (LEfSe) method was used to identify differentially abundant bacteria among the three groups (13). The Kyoto Encyclopedia of Genes and Genomes (KEGG) metabolic pathways were predicted by PICRUSt2 (14). A random forest algorithm was used to determine which bacteria had key roles in distinguishing PA patients from primary hypertension patients and healthy controls. Spearman correlation analysis was performed to demonstrate the relationship between gut microbiota and clinical characteristics.

### Statistical Analysis

Qualitative parameters are presented as numbers and percentages. Quantitative parameters were reported as the mean and standard deviation if they were symmetrically distributed. Otherwise, they are shown as the median and interquartile range. Fisher’s exact test was used to examine qualitative parameters. Student’s *t*-test, analysis of variance (ANOVA), Mann–Whitney *U* test, and Kruskal–Wallis test were applied for testing quantitative parameters. We performed all the statistical analyses in R (version 3.6.3) (R Project for Statistical Computing, www.r-project.org).

## Results

### General Characteristics of Healthy Controls, Primary Hypertension Patients, and PA Patients

A total of 65 participants were enrolled in our study, consisting of 13 PA patients, 26 sex-matched primary hypertension patients, and 26 sex-matched healthy controls (**Table 1**). The mean ages of the control group (50.1 years) and the PA group (46.4 years) were not significantly different (*p* = 0.196), while they were both lower than that of the hypertension group (56.9 years) (*p* < 0.05). BMI was higher in PA patients (24.5) than in healthy controls (23.5) (*p* = 0.043), while it was not significantly different between the PA group and the hypertension group (*p* = 0.126). PA patients had higher DBP than primary hypertension patients (*p* < 0.001), while their SBPs were not significantly different (*p* = 0.151). The blood potassium level of the PA group was lower than those of the hypertension and PA groups (*p* < 0.001). More PA patients had DM than healthy controls (30.8% *vs.* 3.8%, *p* = 0.018). However, the percentages of DM were not significantly different between PA patients and primary hypertension patients (30.8% *vs.* 26.9%, *p* = 0.801). History of smoking and drinking, blood glucose, and blood lipid parameters were not different between the three groups (*p* > 0.05).

**Table 1:** Demographic and clinical characteristics of study participants in control, primary hypertension, and PA patients.

Parameters	PA (*n* = 13)	Primary hypertension (*n* = 26)	Control (*n* = 26)	*p*-value (PA *vs.* primary hypertension *vs.* control)	*p*-value (PA *vs.* control)	*p*-value (PA *vs.* primary hypertension)	*p*-value (primary hypertension *vs.* control)
Sex				1.000 (a)	1.000 (a)	1.000 (a)	1.000 (a)
Male	8 (61.5%)	16 (61.5%)	16 (61.5%)				
Female	5 (38.5%)	10 (38.5%)	10 (38.5%)				
Age (years)	46.4 (11.8)	56.9 (6.9)	50.1 (6.0)	<0.001 (b)	0.196 (b)	0.001 (b)	<0.001 (b)
BMI (kg/m^2^)	24.5 (6.4)	24.1 (5.1)	23.5 (3.5)	0.124 (c)	0.043 (c)	0.126 (c)	0.558 (c)
SBP (mmHg)	147.9 (13.8)	140.9 (14.3)	122.7 (13.2)	<0.001 (b)	<0.001 (b)	0.151 (b)	<0.001 (b)
DBP (mmHg)	99.2 (12.2)	83.2 (8.2)	77.7 (9.6)	<0.001 (b)	<0.001 (b)	<0.001 (b)	0.031 (b)
Aldosterone–renin ratio	426.2 (709.3)	6.1 (7.6)	–	–	–	<0.001 (c)	–
Blood potassium (mmol/L)	3.20 (0.22)	3.93 (0.22)	4.34 (0.37)	<0.001 (b)	<0.001 (b)	<0.001 (b)	<0.001 (b)
Blood glucose (mmol/L)	5.42 (1.33)	5.35 (0.95)	4.88 (0.65)	0.053 (c)	0.036 (c)	0.964 (c)	0.019 (c)
Triglyceride (mmol/L)	0.96 (2.18)	1.45 (0.96)	1.41 (0.41)	0.231 (c)	0.126 (c)	0.136 (c)	0.641 (c)
Cholesterol (mmol/L)	4.35 (0.81)	4.41 (1.02)	4.92 (0.96)	0.101 (b)	0.077 (b)	0.864 (b)	0.070 (b)
HDL (mmol/L)	1.27 (0.33)	1.25 (0.28)	1.26 (0.23)	0.973 (b)	0.924 (b)	0.839 (b)	0.868 (b)
LDL (mmol/L)	2.62 (0.64)	2.54 (0.89)	2.64 (0.81)	0.899 (b)	0.939 (b)	0.772 (b)	0.671 (b)
DM				0.036 (a)	0.035 (a)	1.000 (a)	0.050 (a)
Yes	4 (30.8%)	7 (26.9%)	1 (3.8%)				
No	9 (69.2%)	19 (73.1%)	25 (96.2%)				
Smoking				0.835 (a)	0.719 (a)	0.714 (a)	1.000 (a)
Yes	3 (23.1%)	9 (34.6%)	8 (30.8%)				
No	10 (76.9%)	17 (65.4%)	18 (69.2%)				
Drinking				1.000 (a)	1.000 (a)	1.000 (a)	1.000 (a)
Yes	4 (30.8%)	9 (34.6%)	8 (30.8%)				
No	9 (69.2%)	17 (65.4%)	18 (69.2%)				

(a) Fisher’s exact test, (b) Student’s t-test, (c) Mann–Whitney U test.

PA, primary aldosteronism; BMI, body mass index; SBP, systolic blood pressure; DBP, diastolic blood pressure; HDL, high-density lipoprotein; LDL, low-density lipoprotein; DM, diabetes mellitus.

PA patients used many types of antihypertensive drugs before surgery, including angiotensin-converting enzyme inhibitors, angiotensin receptor blockers, alpha-blockers, beta-blockers, calcium channel blockers, diuretics, and mineralocorticoid receptor antagonists, where only one PA patient used spironolactone. The aldosterone–renin ratio of PA patients was much higher than that of primary hypertension patients (426.2 *vs.* 6.1, *p* < 0.001).

### Diversity of the Gut Microbiota in Healthy Controls, Primary Hypertension Patients, and PA Patients

**Figure 1:**
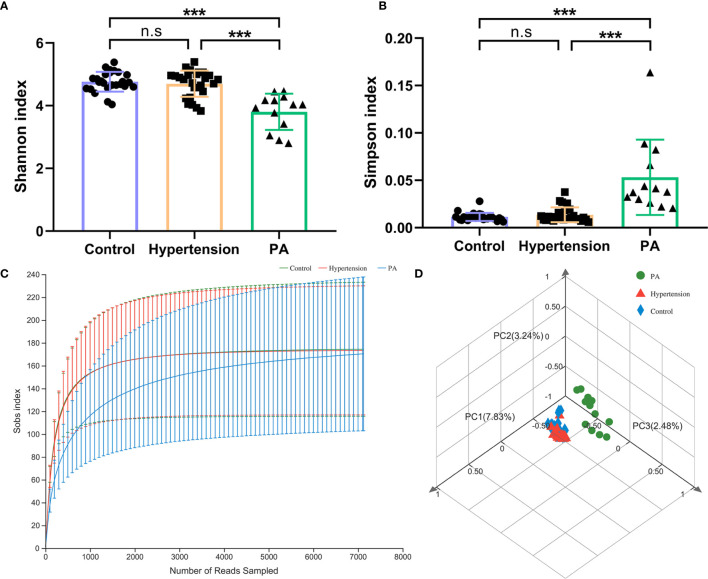
Diversity of the gut microbiota in primary aldosteronism (PA) patients, primary hypertension patients, and healthy controls. **(A)** Shannon index and **(B)** Simpson index of gut microbiota. **(C)** Rarefaction curves of all the samples in the three groups. The horizontal axis shows the number of sequences obtained by sequencing the 16S rRNA gene. The vertical axis shows the number of genera. **(D)** PCoA of gut microbiota by Bray–Curtis distance. ***p < 0.001. n.s, not significant.

Healthy controls and primary hypertension patients had a higher Shannon index and a lower Simpson index than PA patients (*p* < 0.001). This result indicated that the within-sample alpha diversity level of the gut microbiota of PA patients was lower than that of healthy controls and primary hypertension patients (**Figures 1A, B** and **Supplementary Table 1**). The rarefaction curves reached the saturation plateau, and the coverage index ranged from 0.9975 to 0.9998, which indicated that the sequencing depth for the Sobs index was sufficient (**Figure 1C**).

PCoA by Bray–Curtis distance revealed an asymmetrical distribution of gut microbiota composition between the control, hypertension, and PA groups (**Figure 1D**). The ANOSIM test (*R* = 0.599, *p* = 0.001) and PERMANOVA test (*R* ^2^ = 0.093, *p* = 0.001) both verified that there were significant differences in bacterial composition between the three groups.

### Taxonomic Analysis of Microbiota Composition Between Healthy Controls, Primary Hypertension Patients, and PA Patients

**Figure 2:**
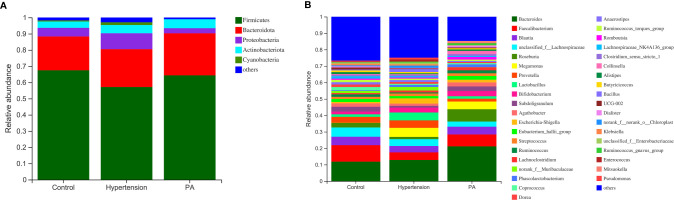
Distribution of gut microbiota between primary aldosteronism (PA) patients, primary hypertension patients, and healthy controls. Relative abundances of the major bacteria at the **(A)** phylum and **(B)** genus levels.

At the phylum level (**Figure 2A**), Firmicutes (mean relative abundance, 62.4%) was the most abundant bacteria in all analyzed samples, followed by Bacteroidota (22.9%), Proteobacteria (6.7%), and Actinobacteria (4.7%). The Firmicutes/Bacteroidetes ratio of the gut microbiota was higher in healthy controls (3.23) than in primary hypertension patients (2.45) and PA patients (2.48). At the genus level (**Figure 2B**), *Bacteroides* was the most common genus, whose percentage in the gut microbiota was much higher in PA patients (21.1%) than in primary hypertension patients (12.9%, *p* = 0.043) and healthy controls (11.8%, *p* = 0.032). The second most common genus was *Faecalibacterium*, whose percentage in the gut microbiota was higher in healthy controls (10.0%) than in primary hypertension patients (4.6%, *p* = 0.011) and PA patients (7.3%, *p* = 0.038).

**Figure 3:**
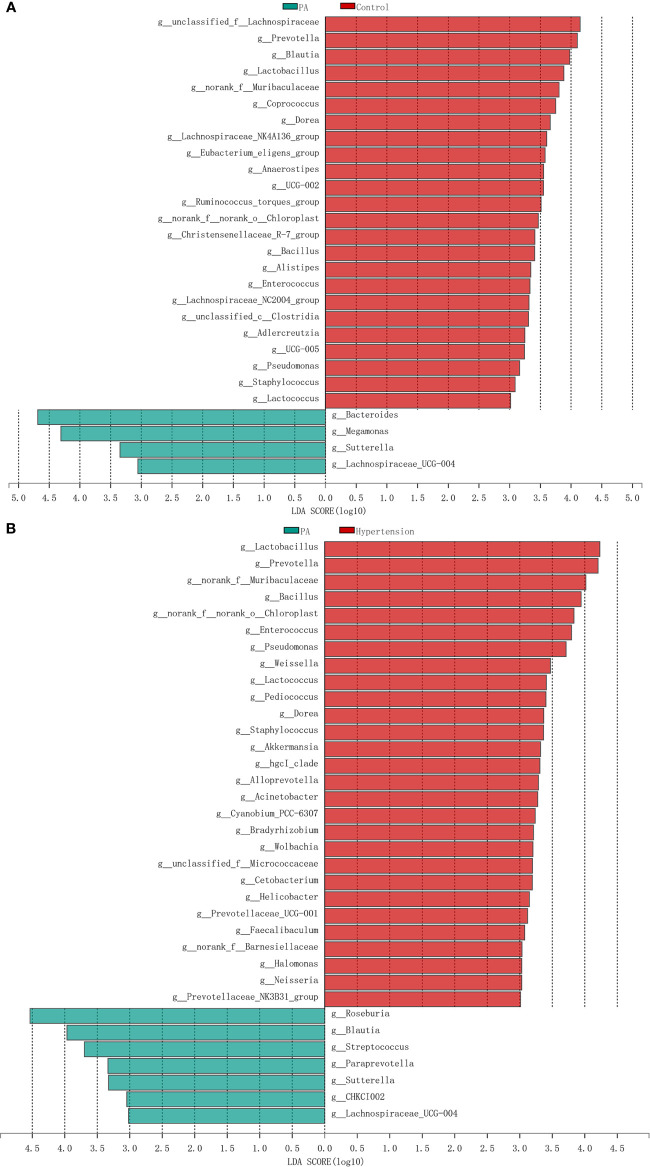
Differences in intestinal bacteria between primary aldosteronism (PA) patients, primary hypertension patients, and healthy controls. **(A)** Significantly different genera between PA patients and healthy controls. Red bars are genera with higher relative abundances in healthy controls. Green bars are genera with higher relative abundances in PA patients. **(B)** Significantly different genera between PA patients and primary hypertension patients. Red bars are genera with higher relative abundances in primary hypertension patients. Green bars are genera with higher relative abundances in PA patients.

LEfSe analysis was used to determine the bacterial genera with significant differences between healthy controls, primary hypertension patients, and PA patients. Compared with PA patients, there were 28 and 35 genera with significantly different relative abundances in healthy controls and primary hypertension patients, respectively (**Figures 3A, B**). For example, PA patients had less *Prevotella* (*p* = 0.018), *Blautia* (*p* = 0.008), *Lactobacillus* (*p* < 0.003), *Coprococcus* (*p* = 0.007), *Eubacterium eligens group* (*p* = 0.004), *Anaerostipes* (*p* = 0.006), *Ruminococcus torques group* (*p* < 0.032), and *Enterococcus* (*p* = 0.006) and more *Bacteroides* (*p* = 0.032), *Megamonas* (*p* = 0.001), and *Sutterella* (*p* = 0.001) than healthy controls. Similarly, the relative abundances of *Lactobacillus* (*p* < 0.001), *Prevotella* (*p* < 0.001), *Weissella* (*p* < 0.001), *Lactococcus* (*p* < 0.001), and *Akkermansia* (*p* < 0.001) were higher in primary hypertension patients, while those of *Roseburia* (*p* = 0.003), *Streptococcus* (*p* = 0.024), *Paraprevotella* (*p* = 0.011), and *Sutterella* (*p* = 0.016) were higher in PA patients. Genera with significant differences between the control and hypertension groups are shown in **Supplementary Figure 1**.

**Figure 4:**
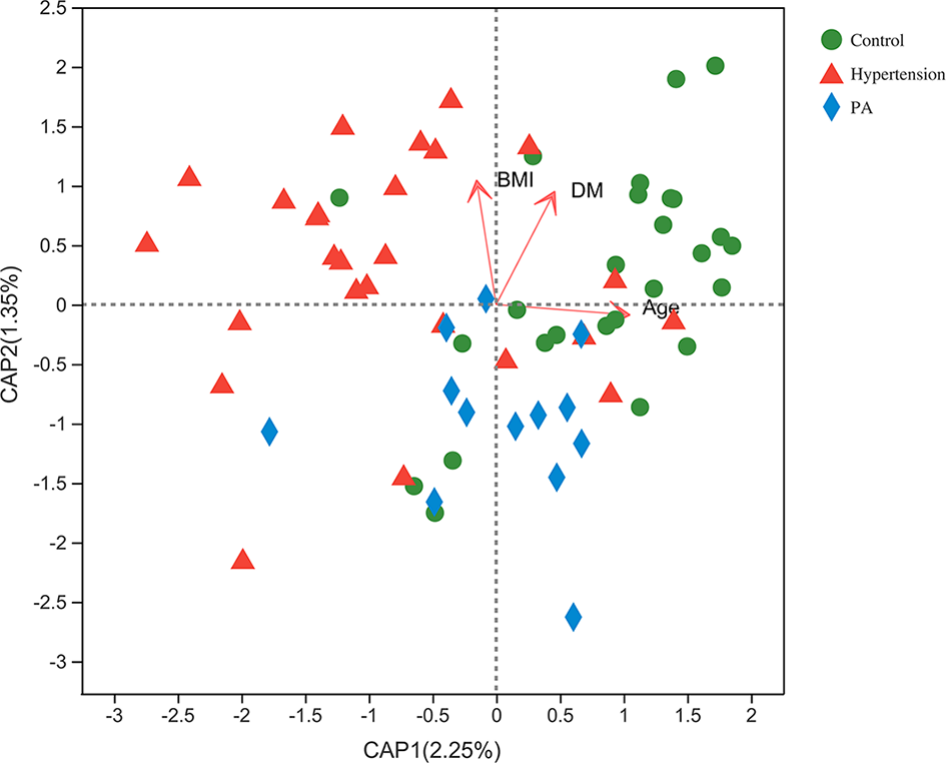
Bray–Curtis distance-based redundancy analysis (dbRDA) of the gut microbiota and explanatory factors in primary aldosteronism (PA) patients, primary hypertension patients, and healthy controls.

We performed a Bray–Curtis distance-based redundancy analysis (dbRDA) to determine the additional effects of age, BMI, and DM on the gut microbiota (**Figure 4**). The envfit function test showed that age (*p* = 0.001), BMI (*p* = 0.001), and DM (*p* = 0.001) were significant explanatory variables (**Supplementary Table 2**).

To eliminate the possible confounding effects of DM and BMI, we excluded participants with DM. BMI and blood glucose were not significantly different between healthy controls, primary hypertension patients, and PA patients after removing DM patients (*p* > 0.05) (**Supplementary Table 3**). LEfSe analysis revealed that the genera with significant differences between the three groups were similar to those before deleting participants with DM (**Supplementary Figure 2**). After deleting DM patients, the relative abundance of *Eubacterium* (*p* = 0.017) was higher in PA patients than in healthy controls. PA patients also had more *Bacteroides* (*p* = 0.013), *Phascolarctobacterium* (*p* = 0.024), *Moryella* (*p* = 0.009), and *Eubacterium fissicatena group* (*p* = 0.036) than primary hypertension patients after deleting DM patients.

### Metabolic Function of Gut Microbiota

To evaluate the gene information of gut microbiota, metabolic predictions were achieved using PICRUSt2. The relative abundance of metabolic pathways between the three groups was compared by the Wilcoxon rank sum test. The Benjamini–Hochberg false discovery rate (FDR)-adjusted *p*-values were also calculated. Compared with healthy controls, PA patients had more lipopolysaccharide biosynthesis (FDR-adjusted *p*-value = 0.017), galactose metabolism (FDR-adjusted *p*-value = 0.001), pentose and glucuronate interconversions (FDR-adjusted *p*-value = 0.004), amino sugar and nucleotide sugar metabolism (FDR-adjusted *p*-value = 0.001), fructose and mannose metabolism (FDR-adjusted *p*-value = 0.004), starch and sucrose metabolism (FDR-adjusted *p*-value = 0.002), and arginine and proline metabolism (FDR-adjusted *p*-value = 0.006) (**Supplementary Table 4**). The relative abundance of tryptophan metabolism (FDR-adjusted *p*-value = 0.010) was higher in primary hypertension patients, while those of galactose metabolism (FDR-adjusted *p*-value = 0.005), insulin resistance (FDR-adjusted *p*-value = 0.001), starch and sucrose metabolism (FDR-adjusted *p*-value = 0.003), amino sugar and nucleotide sugar metabolism (FDR-adjusted *p*-value = 0.011), pentose and glucuronate interconversions (FDR-adjusted *p*-value = 0.018), and insulin signaling pathway (FDR-adjusted *p*-value = 0.041) were higher in PA patients (**Supplementary Table 5**). **Supplementary Table 6** shows the metabolic pathways with significant differences between the control and hypertension groups.

### Bacteria Distinguishing PA Patients From Primary Hypertension Patients and Healthy Controls

**Figure 5:**
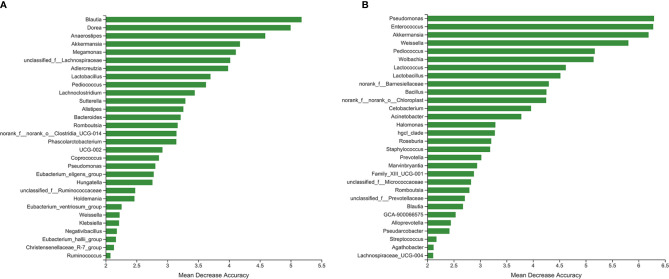
The top 30 genera with great importance in distinguishing primary aldosteronism (PA) patients from **(A)** healthy controls and **(B)** primary hypertension patients.

We used random forest algorithm to determine which bacteria had key roles in distinguishing PA patients from healthy controls. **Figure 5A** shows the top 30 genera with the greatest importance, some of which were also screened out by LEfSe analysis, such as *Blautia*, *Anaerostipes*, *Megamonas*, and *Lactobacillus*. The area under the curve (AUC) of the receiver operating characteristic curve was 0.8173 using the random forest model in combination with leave-one-out cross-validation. Similarly, **Figure 5B** presents the top 30 genera with the greatest importance in distinguishing PA patients from primary hypertension patients. The following genera were also detected in the LEfSe analysis: *Pseudomonas*, *Enterococcus*, *Akkermansia*, *Weissella*, and *Pediococcus*. The AUC of the receiver operating characteristic curve was 0.9231.

### Association Between Clinical Characteristics and Gut Microbiota

**Figure 6:**
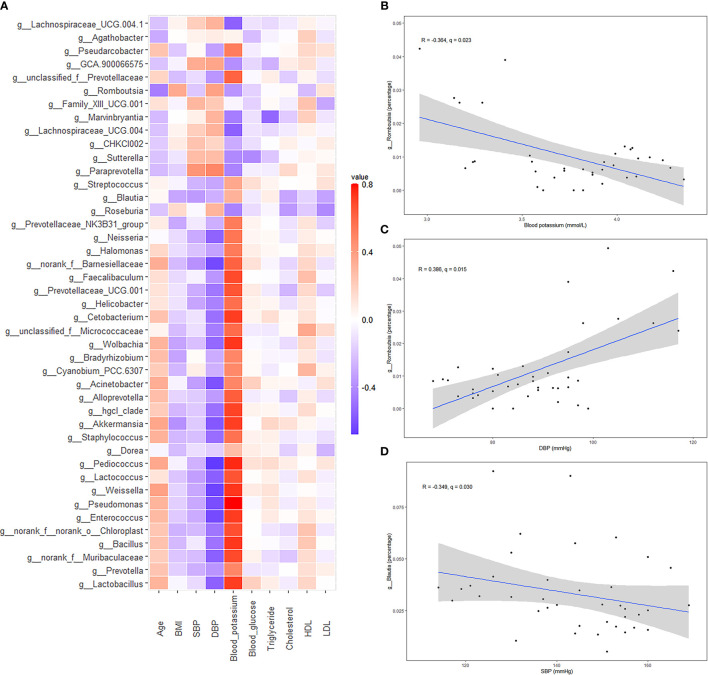
Spearman correlation analysis of different bacteria [primary aldosteronism (PA) patients *vs.* primary hypertension patients] and clinical characteristics. **(A)** Heatmap showing the correlation between different bacteria and clinical characteristics. **(B)** Scatter plot graphs showing the correlation between blood potassium and *Romboutsia*. **(C)** Scatter plot graphs showing the correlation between diastolic blood pressure (DBP) and *Romboutsia*. **(D)** Scatter plot graphs showing the correlation between systolic blood pressure (SBP) and *Blautia*.

Heatmap analysis (**Figure 6A**) showed that the bacteria with lower relative abundances in the PA group than in the hypertension group were positively correlated with blood potassium and negatively correlated with SBP and DBP. We specifically drew scatter plot graphs of genera with high percentages in the gut microbiota (**Figures 6B–D**). Blood potassium was negatively correlated with the relative abundance of *Romboutsia* (*R* = −0.364, *q* = 0.023). In addition, DBP was positively correlated with *Romboutsia* (*R* = 0.386, *q* = 0.015). SBP was negatively correlated with *Blautia* (*R* = −0.349, *q* = 0.030).

## Discussion

This study is the first to demonstrate the characteristics of the gut microbiota in PA patients. The gut microbiota has been reported to be associated with metabolic diseases, such as DM and obesity (6, 7). DM and obesity are more common in PA patients than in healthy people or primary hypertension patients (2, 3). Our study showed that DM was more common in PA patients than in healthy controls. The BMI of PA patients was higher than that of healthy controls. In addition, the composition of the gut microbiota and its metabolic pathways of PA patients were also significantly different from those of healthy controls and primary hypertension patients.

We hypothesized that PA may contribute to the development of DM and obesity *via* gut microbiota. On the one hand, the alpha diversity of the gut microbiota in PA patients was lower than that in primary hypertension patients and healthy controls, which meant that the richness of the gut microbiota of PA patients was disturbed. Folz et al. reported that high-sodium and low-potassium intake could interact with gut microbiota to affect endocrine homeostasis (15). No study has clearly demonstrated the relationship between aldosterone and gut microbiota. It was reasonable to assume that PA is responsible for the changes in gut microbiota. On the other hand, the gut microbiota also affects DM and obesity by regulating inflammation, gut permeability, glucose metabolism, fatty acid oxidation, synthesis and energy expenditure, and the interaction of gut bacteria (6). The richness of gut microbiota in metabolic disease patients decreased (16). The Firmicutes/Bacteroidetes ratio was reported to be lower in individuals with obesity (17).

We found that the relative abundances of *Prevotella*, *Blautia*, *Coprococcus*, *Anaerostipes*, and *Ruminococcus* were lower in the gut microbiota of PA patients than in healthy controls and primary hypertension patients. All these bacteria could produce SCFAs (18–21). SCFAs, the energy sources of enterocytes, could maintain the intestinal epithelial barrier and decrease the permeability of the gut, circulating lipopolysaccharides, and systemic inflammation. SCFAs could also reduce inflammation *via* GPR41/43 (22, 23). In addition, *Lactobacillus* was more abundant in the gut microbiota of healthy controls and primary hypertension patients than in PA patients. Several studies have already demonstrated that the administration of *Lactobacillus* could alleviate hyperglycemia in diabetic rats (6, 24, 25). We also found that *Lactococcus* and *Enterococcus* were present in lower abundances in PA patients. Lilia et al. reported that consumption of fermented milk with *Lactococcus lactis* had a blood pressure-lowering effect on prehypertensive subjects (26). Anne et al. found that vertical sleeve gastrectomy reduced body mass and blood pressure and increased the relative abundance of *Enterococcus* in the gut microbiota of mice (27). In contrast, PA patients had more *Megamonas*, *Sutterella*, and *Streptococcus* than healthy controls and primary hypertension patients, genera that are associated with inflammation (28–30). *Weissella* and *Akkermansia*, which were present in lower abundances in PA patients than in primary hypertension patients, were shown to have anti-inflammatory potential (31, 32). PA patients had a higher relative abundance of lipopolysaccharide biosynthesis in the gut microbiota than healthy controls. Lipopolysaccharides are bacterial surface glycolipids produced by gram-negative bacteria, which can induce inflammatory reactions (33). It was reported that inflammatory factors could alter glucose tolerance and insulin sensitivity (34). Individuals with obesity had more *Megamonas* (7). Compared with healthy controls and primary hypertension patients, PA patients had more pathways participating in sugar metabolism, such as starch and sucrose metabolism, fructose and mannose metabolism, amino sugar and nucleotide sugar metabolism, pentose and glucuronate interconversions, arginine and proline metabolism, and galactose metabolism. These factors may result in more glucose in the gut and absorption into the blood. In addition, some amino acid metabolism pathways were also significantly different between the PA and hypertension groups. For example, tryptophan metabolism was lower in PA patients. Tryptophan metabolism could produce some metabolites, such as indole-3-ethanol, indole-3-pyruvate, and indole-3-aldehyde, which are essential in maintaining intestinal barrier function (35). This evidence indicated that PA-associated dysbiosis of gut microbiota in SCFAs, sugar and amino acid metabolism, and inflammation were associated with metabolic disorders.

Previous studies regarded hypokalemia as a risk factor for DM. They thought that hypokalemia may decrease insulin secretion and induce insulin resistance, followed by dysbiosis of glucose metabolism (36). We found that *Romboutisa* and *Bacteroides* were more abundant in PA patients than in primary hypertension patients. *Romboutisa* was also negatively correlated with blood potassium. Some studies found that *Romboutsia* was positively associated with obesity and lipid metabolism (37, 38). *Bacteroides* was also found to be increased after the higher-fat diet intervention (39) and to be more abundant in patients with DM among the Chinese population (40). This evidence indicated that gut microbiota may also play an important role in hypokalemia-related disorders of lipid and glucose metabolism, a possibility that needs further exploration.

Our study first revealed the characteristics of the gut microbiota of PA patients and identified some bacteria and metabolic pathways that may be associated with DM and obesity. We believe that the knowledge gained from this study will be important in shaping the framework and platform for broader research on the gut microbiota of PA patients in the future.

The study showed that there was a significant difference in the mean age between PA patients and primary hypertension patients. Several studies have revealed that age is a factor influencing the gut microbiota (41–44). As people age, key changes in gut microbiota include compositional instability, reduced overall diversity, and an increase in proinflammatory opportunistic pathogens. In infants, *Bifidobacterium* is the most dominant genus in the gut microbiota. Biagi et al. examined the fecal microbiome of young adults (22–48 years old), elderly adults (65–75 years old), and semisupercentenarians (105–109 years old) in an Italian population (45). They found that the fecal microbiota in all age groups was dominated by just three families, including Bacteroidaceae, Lachnospiraceae, and Ruminococcaceae. However, the relative abundance of bacteria belonging to these families decreased with age. An independent study of Chinese centenarians reported similar results (46). In addition, they both found that longevity increased microbial community richness and the abundance of subdominant but health-related bacterial genera and families, such as *Oscillospira*, Christensenellaceae, *Akkermansia*, and *Bifidobacterium*. The diversity of gut microbiota continues to increase until the age of 3 years, remains stable afterwards, and then declines during senescence (44). The gut microbiota of elderly people is reduced in beneficial microbes, such as SCFA producers, and enriched in proinflammatory microbes (42, 44).

This study had some limitations. First, almost all PA and primary hypertension patients had received different antihypertensive medications before recruitment. It was unclear whether these drugs affected the composition and metabolism of gut microbiota. Second, the effects of some factors on the gut microbiota could not be ignored, such as DM, aging, use of antihypertensive drugs, and salt intake (47). Third, the sample size of the study was relatively small and some characteristics were heterogeneous between groups, which may contribute to biases. Further case–control studies should have larger sample sizes; match factors that will potentially affect gut microbiota, such as age and dietary habits; and measure the 24-h urinary electrolytes to investigate the role of gut microbiota in metabolic disorders in primary aldosteronism patients and reduce analysis bias.

In conclusion, the alteration of gut microbiota in PA patients, especially bacteria and pathways involved in inflammation, SCFAs, and sugar metabolism, may be associated with chronic metabolic disorders.

## Data Availability Statement

The datasets presented in this study can be found in online repositories. The names of the repository/repositories and accession number(s) can be found below: NCBI accession: PRJNA728662.

## Ethics Statement

The studies involving human participants were reviewed and approved by the West China Hospital, Sichuan University Medical Research Ethics Committee. The patients/participants provided their written informed consent to participate in this study.

## Author Contributions

YL and QJ conceived and designed the study, collected and analyzed the data, and wrote the manuscript. ZL, SS, and JA analyzed the data. YZ and LZ reviewed and edited the manuscript. All authors contributed to the article and approved the submitted version.

## Funding

This work was supported by 1.3.5 Project for Disciplines of Excellence, West China Hospital, Sichuan University (ZY2016104).

## Conflict of Interest

The authors declare that the research was conducted in the absence of any commercial or financial relationships that could be construed as a potential conflict of interest.

## Publisher’s Note

All claims expressed in this article are solely those of the authors and do not necessarily represent those of their affiliated organizations, or those of the publisher, the editors and the reviewers. Any product that may be evaluated in this article, or claim that may be made by its manufacturer, is not guaranteed or endorsed by the publisher.
