# Supplementary material for: Alteration of Gut Microbiota Relates to Metabolic Disorders in Primary Aldosteronism Patients
Source: Front Endocrinol (Lausanne). 2021 Aug 17;12:667951. doi: 10.3389/fendo.2021.667951 (PMC8415980; doi:10.3389/fendo.2021.667951)
Supplement: Supplementary file 1 [file DataSheet_1.docx]

**Supplementary Materials**

**Supplementary Table 1. Alpha diversity indices of the gut microbiota in PA patients, primary hypertension patients and healthy controls.**

| **Alpha diversity index** | **PA** | |  | **Primary hypertension** | |  | **Control** | |  | **q value** | | |
| --- | --- | --- | --- | --- | --- | --- | --- | --- | --- | --- | --- | --- |
|  | **Mean** | **SD** |  | **Mean** | **SD** |  | **Mean** | **SD** |  | **PA-Control** | **PA-Primary hypertension** | **Primary hypertension-Control** |
| **Shannon** | 3.805 | 0.579 |  | 4.700 | 0.419 |  | 4.764 | 0.315 |  | < 0.001 | < 0.001 | 1.000 |
| **Simpson** | 0.053 | 0.040 |  | 0.014 | 0.008 |  | 0.011 | 0.004 |  | < 0.001 | < 0.001 | 1.000 |

PA, primary aldosteronism; SD, standard deviation.

**Supplementary Table 2. Bray–Curtis distance-based redundancy analysis (dbRDA) envfit analysis of the correlation between the gut microbiota and explanatory variables in PA patients, primary hypertension patients and healthy controls.**

|  | **RDA1** | **RDA2** | **r^2^** | **p value** |
| --- | --- | --- | --- | --- |
| **Age** | 0.9965 | -0.0831 | 0.3598 | 0.001 |
| **BMI** | -0.1452 | 0.9894 | 0.4685 | 0.001 |
| **DM** | 0.4354 | 0.9002 | 0.2593 | 0.001 |

PA, primary aldosteronism.

RDA1 and RDA2 are the cosines of the angles between the arrows of the explanatory variables and the sorting axes.

**Supplementary Table 3. Demographic and clinical characteristics of the study participants without DM in PA patients, primary hypertension patients and healthy controls.**

| **Parameters** | | **PA (n = 9)** | | **Primary hypertension (n = 19)** | | | **Control (n = 25)** | | **p value**  **(PA vs. Primary hypertension vs. Control)** | **p value (PA vs. Control)** | | **p value (PA vs. Primary hypertension)** | | **p value (Primary hypertension vs. Control)** | |
| --- | --- | --- | --- | --- | --- | --- | --- | --- | --- | --- | --- | --- | --- | --- | --- |
| **Sex** | |  | |  |  | | 1.000 (a) | | | 1.000 (a) | | 1.000 (a) | | 1.000 (a) |  |
| Male | | 5 (55.6%) | | 11 (57.9%) | 15 (60%) | |  | | |  | |  | |  |  |
| Female | | 4 (44.4%) | | 8 (42.1%) | 10 (40%) | |  | | |  | |  | |  |  |
| **Age (years)** | | 45.0 (14.0) | | 56.7 (6.4) | 49.8 (5.4) | | 0.001 (b) | | | 0.161 (b) | | 0.005 (b) | | 0.001 (b) |  |
| **BMI (kg/m2)** | | 23.7 (5.6) | | 24.0 (4.6) | 23.4 (3.2) | | 0.408 (c) | | | 0.231 (c) | | 0.223 (c) | | 0.963 (c) |  |
| **SBP (mmHg)** | | 147.3 (16.4) | | 142.5 (14.4) | 123.4 (13.0) | | < 0.001 (b) | | | < 0.001 (b) | | 0.433 (b) | | < 0.001 (b) |  |
| **DBP (mmHg)** | | 100.3 (14.2) | | 83.4 (8.8) | 78.0 (9.7) | | < 0.001 (b) | | | < 0.001 (b) | | 0.001 (b) | | 0.062 (b) |  |
| **Aldosterone-renin ratio** | | 531.8 (1104.1) | | 6.0 (5.6) | - | | - | | | - | | < 0.001 (c) | | - |  |
| **Blood potassium (mmol/L)** | | 3.22 (0.26) | | 3.93 (0.22) | 4.33 (0.38) | | < 0.001 (b) | | | < 0.001 (b) | | < 0.001 (b) | | < 0.001 (b) |  |
| **Blood glucose (mmol/L)** | | 4.92 (1.62) | | 5.12 (0.85) | 4.90 (0.69) | | 0.354 (c) | | | 0.618 (c) | | 0.923 (c) | | 0.123 (c) |  |
| **Triglyceride (mmol/L)** | | 0.87 (0.39) | | 1.43 (1.60) | 1.38 (0.39) | | 0.017 (c) | | | 0.004 (c) | | 0.019 (c) | | 0.610 (c) |  |
| **Cholesterol (mmol/L)** | | 4.31 (0.97) | | 4.43 (1.08) | 4.82 (0.84) | | 0.263 (b) | | | 0.146 (b) | | 0.783 (b) | | 0.186 (b) |  |
| **HDL (mmol/L)** | | 1.31 (0.33) | | 1.27 (0.31) | 1.26 (0.24) | | 0.887 (b) | | | 0.603 (b) | | 0.750 (b) | | 0.881 (b) |  |
| **LDL (mmol/L)** | | 2.66 (0.76) | | 2.59 (0.90) | 2.55 (0.70) | | 0.942 (b) | | | 0.708 (b) | | 0.843 (b) | | 0.883 (b) |  |
| **Smoking** | |  | |  |  | | 0.559 (a) | | | 0.386 (a) | | 0.371 (a) | | 1.000 (a) |  |
| Yes | | 1 (11.1%) | | 6 (31.6%) | 8 (32.0%) | |  | | |  | |  | |  |  |
| No | | 8 (88.9%) | | 13 (68.4%) | 17 (68.0%) | |  | | |  | |  | |  |  |
| **Drinking** | |  | |  |  | | 0.926 (a) | | | 0.692 (a) | | 1.000 (a) | | 1.000 (a) |  |
| Yes | | 2 (22.2%) | | 6 (31.6%) | 8 (32.0%) | |  | | |  | |  | |  |  |
| No | | 7 (77.8%) | | 13 (68.4%) | 17 (68.0%) | |  | | |  | |  | |  |  |

DM, Diabetes mellitus; PA, primary aldosteronism; BMI, body mass index; SBP, systolic blood pressure; DBP, diastolic blood pressure; HDL, high-density lipoprotein; LDL, low-density lipoprotein.

(a) Fisher's exact test, (b) Student’s t test, (c) Mann-Whitney test.

**Supplementary Table 4: Metabolic pathways with statistical difference between PA patients and healthy controls.**

| **No.** | **Metabolic pathways** | **p value** | **FDR-adjusted p value** | **Fold Change (PA/Control)** |
| --- | --- | --- | --- | --- |
| 1 | Penicillin and cephalosporin biosynthesis | < 0.001 | 0.001 | 1.820 |
| 2 | Protein digestion and absorption | 0.019 | 0.047 | 1.663 |
| 3 | Lysosome | 0.007 | 0.021 | 1.602 |
| 4 | Lipopolysaccharide biosynthesis | 0.005 | 0.017 | 1.602 |
| 5 | Phospholipase D signaling pathway | 0.009 | 0.025 | 1.591 |
| 6 | Axon regeneration | 0.009 | 0.025 | 1.581 |
| 7 | Choline metabolism in cancer | 0.008 | 0.023 | 1.573 |
| 8 | Other glycan degradation | 0.007 | 0.021 | 1.481 |
| 9 | Phosphotransferase system (PTS) | 0.002 | 0.009 | 1.426 |
| 10 | Phenylpropanoid biosynthesis | 0.002 | 0.007 | 1.353 |
| 11 | Glycosphingolipid biosynthesis - globo and isoglobo series | 0.005 | 0.015 | 1.341 |
| 12 | Primary bile acid biosynthesis | 0.001 | 0.006 | 1.322 |
| 13 | Phosphonate and phosphinate metabolism | 0.001 | 0.004 | 1.319 |
| 14 | Sphingolipid metabolism | 0.005 | 0.017 | 1.318 |
| 15 | Galactose metabolism | < 0.001 | 0.001 | 1.291 |
| 16 | Isoquinoline alkaloid biosynthesis | < 0.001 | 0.002 | 1.277 |
| 17 | Pentose and glucuronate interconversions | 0.001 | 0.004 | 1.267 |
| 18 | beta-Lactam resistance | < 0.001 | 0.001 | 1.265 |
| 19 | Biofilm formation - Vibrio cholerae | < 0.001 | 0.002 | 1.254 |
| 20 | Cyanoamino acid metabolism | 0.001 | 0.003 | 1.254 |
| 21 | Amino sugar and nucleotide sugar metabolism | < 0.001 | 0.001 | 1.249 |
| 22 | Cationic antimicrobial peptide (CAMP) resistance | < 0.001 | 0.003 | 1.246 |
| 23 | Tropane, piperidine and pyridine alkaloid biosynthesis | < 0.001 | 0.001 | 1.223 |
| 24 | Biosynthesis of vancomycin group antibiotics | 0.001 | 0.004 | 1.219 |
| 25 | Fructose and mannose metabolism | 0.001 | 0.004 | 1.205 |
| 26 | Neomycin, kanamycin and gentamicin biosynthesis | 0.009 | 0.025 | 1.204 |
| 27 | Vitamin B6 metabolism | 0.001 | 0.004 | 1.201 |
| 28 | Starch and sucrose metabolism | < 0.001 | 0.002 | 1.199 |
| 29 | Citrate cycle (TCA cycle) | 0.002 | 0.006 | 1.192 |
| 30 | Phosphatidylinositol signaling system | 0.002 | 0.007 | 1.190 |
| 31 | Biotin metabolism | < 0.001 | 0.002 | 1.187 |
| 32 | Epithelial cell signaling in Helicobacter pylori infection | < 0.001 | 0.003 | 1.187 |
| 33 | Protein processing in endoplasmic reticulum | 0.021 | 0.050 | 1.180 |
| 34 | Glyoxylate and dicarboxylate metabolism | 0.001 | 0.004 | 1.178 |
| 35 | Photosynthesis | < 0.001 | 0.001 | 1.163 |
| 36 | Type I diabetes mellitus | 0.002 | 0.008 | 1.163 |
| 37 | Zeatin biosynthesis | 0.002 | 0.007 | 1.162 |
| 38 | Bacterial secretion system | 0.001 | 0.004 | 1.158 |
| 39 | Histidine metabolism | < 0.001 | 0.002 | 1.158 |
| 40 | Selenocompound metabolism | 0.001 | 0.003 | 1.157 |
| 41 | Biofilm formation - Escherichia coli | 0.002 | 0.009 | 1.152 |
| 42 | Monobactam biosynthesis | 0.001 | 0.006 | 1.151 |
| 43 | Carbon fixation pathways in prokaryotes | < 0.001 | 0.003 | 1.148 |
| 44 | Arginine and proline metabolism | 0.002 | 0.006 | 1.148 |
| 45 | One carbon pool by folate | 0.001 | 0.003 | 1.148 |
| 46 | Type II diabetes mellitus | < 0.001 | 0.001 | 1.146 |
| 47 | Novobiocin biosynthesis | < 0.001 | 0.002 | 1.146 |
| 48 | Central carbon metabolism in cancer | < 0.001 | 0.003 | 1.144 |
| 49 | Pyrimidine metabolism | < 0.001 | 0.002 | 1.143 |
| 50 | Plant-pathogen interaction | 0.006 | 0.020 | 1.140 |
| 51 | Polyketide sugar unit biosynthesis | 0.004 | 0.014 | 1.138 |
| 52 | Acarbose and validamycin biosynthesis | 0.008 | 0.023 | 1.137 |
| 53 | Propanoate metabolism | 0.003 | 0.009 | 1.137 |
| 54 | D-Alanine metabolism | < 0.001 | 0.003 | 1.136 |
| 55 | Oxidative phosphorylation | 0.001 | 0.004 | 1.134 |
| 56 | Carbapenem biosynthesis | < 0.001 | 0.003 | 1.134 |
| 57 | Glucosinolate biosynthesis | 0.001 | 0.003 | 1.133 |
| 58 | Streptomycin biosynthesis | 0.007 | 0.021 | 1.131 |
| 59 | Antifolate resistance | 0.002 | 0.007 | 1.131 |
| 60 | Drug metabolism - other enzymes | 0.001 | 0.004 | 1.130 |
| 61 | Antigen processing and presentation | 0.003 | 0.009 | 1.129 |
| 62 | Progesterone-mediated oocyte maturation | 0.003 | 0.009 | 1.129 |
| 63 | Th17 cell differentiation | 0.003 | 0.009 | 1.129 |
| 64 | IL-17 signaling pathway | 0.003 | 0.009 | 1.129 |
| 65 | Prostate cancer | 0.003 | 0.009 | 1.129 |
| 66 | Estrogen signaling pathway | 0.003 | 0.009 | 1.129 |
| 67 | Alanine, aspartate and glutamate metabolism | 0.001 | 0.004 | 1.128 |
| 68 | Purine metabolism | 0.001 | 0.004 | 1.124 |
| 69 | Glycine, serine and threonine metabolism | < 0.001 | 0.003 | 1.123 |
| 70 | Biosynthesis of secondary metabolites | < 0.001 | 0.002 | 1.121 |
| 71 | Proteoglycans in cancer | < 0.001 | 0.003 | 1.121 |
| 72 | Glycolysis / Gluconeogenesis | 0.001 | 0.004 | 1.120 |
| 73 | D-Glutamine and D-glutamate metabolism | < 0.001 | 0.003 | 1.119 |
| 74 | Homologous recombination | 0.001 | 0.004 | 1.119 |
| 75 | Microbial metabolism in diverse environments | 0.001 | 0.004 | 1.117 |
| 76 | Cysteine and methionine metabolism | 0.001 | 0.003 | 1.116 |
| 77 | 2-Oxocarboxylic acid metabolism | 0.006 | 0.018 | 1.115 |
| 78 | RNA degradation | 0.001 | 0.006 | 1.113 |
| 79 | Ribosome biogenesis in eukaryotes | < 0.001 | 0.001 | 1.113 |
| 80 | GABAergic synapse | 0.019 | 0.047 | 1.113 |
| 81 | Protein export | < 0.001 | 0.003 | 1.112 |
| 82 | Pentose phosphate pathway | 0.007 | 0.021 | 1.111 |
| 83 | Quorum sensing | 0.021 | 0.050 | 1.110 |
| 84 | Carbon metabolism | < 0.001 | 0.002 | 1.109 |
| 85 | Nitrogen metabolism | 0.001 | 0.003 | 1.109 |
| 86 | Ribosome | < 0.001 | 0.003 | 1.109 |
| 87 | Butanoate metabolism | 0.002 | 0.007 | 1.109 |
| 88 | Biosynthesis of amino acids | 0.002 | 0.009 | 1.107 |
| 89 | Cell cycle - Caulobacter | 0.001 | 0.004 | 1.106 |
| 90 | Salmonella infection | 0.019 | 0.047 | 1.105 |
| 91 | Lysine biosynthesis | 0.003 | 0.011 | 1.104 |
| 92 | Valine, leucine and isoleucine biosynthesis | 0.016 | 0.041 | 1.104 |
| 93 | Phenylalanine, tyrosine and tryptophan biosynthesis | 0.008 | 0.022 | 1.101 |
| 94 | Carbon fixation in photosynthetic organisms | 0.001 | 0.005 | 1.101 |
| 95 | DNA replication | < 0.001 | 0.003 | 1.100 |
| 96 | HIF-1 signaling pathway | 0.008 | 0.022 | 1.099 |
| 97 | Folate biosynthesis | 0.012 | 0.031 | 1.098 |
| 98 | Riboflavin metabolism | 0.001 | 0.004 | 1.095 |
| 99 | Nicotinate and nicotinamide metabolism | 0.001 | 0.006 | 1.094 |
| 100 | Base excision repair | 0.001 | 0.004 | 1.094 |
| 101 | Sulfur metabolism | 0.019 | 0.047 | 1.093 |
| 102 | Aminoacyl-tRNA biosynthesis | 0.001 | 0.006 | 1.092 |
| 103 | Glycerophospholipid metabolism | 0.004 | 0.013 | 1.091 |
| 104 | Pantothenate and CoA biosynthesis | 0.003 | 0.010 | 1.090 |
| 105 | Vancomycin resistance | 0.013 | 0.033 | 1.090 |
| 106 | Necroptosis | 0.009 | 0.025 | 1.089 |
| 107 | RNA transport | 0.015 | 0.038 | 1.088 |
| 108 | Peptidoglycan biosynthesis | 0.011 | 0.029 | 1.088 |
| 109 | Viral carcinogenesis | 0.001 | 0.004 | 1.087 |
| 110 | Human papillomavirus infection | 0.001 | 0.004 | 1.087 |
| 111 | Nucleotide excision repair | 0.004 | 0.013 | 1.086 |
| 112 | Fluid shear stress and atherosclerosis | 0.003 | 0.009 | 1.086 |
| 113 | Sulfur relay system | 0.011 | 0.029 | 1.084 |
| 114 | Terpenoid backbone biosynthesis | 0.001 | 0.004 | 1.083 |
| 115 | Mismatch repair | 0.002 | 0.009 | 1.082 |
| 116 | Tuberculosis | 0.004 | 0.014 | 1.079 |
| 117 | Fatty acid biosynthesis | 0.002 | 0.007 | 1.078 |
| 118 | Thiamine metabolism | 0.021 | 0.050 | 1.075 |
| 119 | RNA polymerase | 0.015 | 0.038 | 1.074 |
| 120 | Methane metabolism | 0.019 | 0.047 | 1.073 |
| 121 | Pyruvate metabolism | 0.003 | 0.011 | 1.071 |
| 122 | Synthesis and degradation of ketone bodies | 0.002 | 0.009 | 0.623 |
| 123 | Styrene degradation | 0.014 | 0.036 | 0.512 |
| 124 | Renin-angiotensin system | 0.002 | 0.009 | 0.399 |
| 125 | Carotenoid biosynthesis | < 0.001 | 0.003 | 0.090 |
| 126 | Parkinson disease | < 0.001 | 0.003 | 0.040 |
| 127 | Polycyclic aromatic hydrocarbon degradation | 0.001 | 0.005 | 0.037 |
| 128 | Non-alcoholic fatty liver disease (NAFLD) | < 0.001 | 0.001 | 0.031 |
| 129 | Herpes simplex virus 1 infection | < 0.001 | 0.002 | 0.028 |
| 130 | Small cell lung cancer | < 0.001 | 0.001 | 0.026 |
| 131 | Apoptosis - multiple species | < 0.001 | 0.001 | 0.026 |
| 132 | Colorectal cancer | < 0.001 | 0.001 | 0.026 |
| 133 | Epstein-Barr virus infection | < 0.001 | 0.001 | 0.026 |
| 134 | Viral myocarditis | < 0.001 | 0.001 | 0.026 |
| 135 | p53 signaling pathway | < 0.001 | 0.001 | 0.026 |
| 136 | Hepatitis B | < 0.001 | 0.001 | 0.026 |
| 137 | Measles | < 0.001 | 0.001 | 0.026 |
| 138 | Hepatitis C | < 0.001 | 0.001 | 0.026 |
| 139 | Influenza A | < 0.001 | 0.001 | 0.026 |
| 140 | Human cytomegalovirus infection | < 0.001 | 0.001 | 0.026 |
| 141 | Kaposi sarcoma-associated herpesvirus infection | < 0.001 | 0.001 | 0.026 |
| 142 | Human immunodeficiency virus 1 infection | < 0.001 | 0.001 | 0.026 |
| 143 | Toxoplasmosis | < 0.001 | 0.001 | 0.025 |
| 144 | Photosynthesis - antenna proteins | 0.013 | 0.034 | 0.024 |
| 145 | Basal transcription factors | 0.007 | 0.021 | 0.020 |
| 146 | Steroid biosynthesis | < 0.001 | 0.002 | 0.017 |
| 147 | Sesquiterpenoid and triterpenoid biosynthesis | < 0.001 | 0.003 | 0.016 |
| 148 | Vibrio cholerae infection | 0.021 | 0.050 | 0.016 |
| 149 | Sphingolipid signaling pathway | 0.007 | 0.021 | 0.004 |
| 150 | Steroid degradation | 0.003 | 0.012 | 0.004 |
| 151 | Isoflavonoid biosynthesis | 0.012 | 0.032 | 0.001 |
| 152 | Melanogenesis | 0.008 | 0.022 | 0.001 |
| 153 | Parathyroid hormone synthesis, secretion and action | 0.001 | 0.005 | 0.001 |
| 154 | Systemic lupus erythematosus | 0.012 | 0.032 | 0.001 |
| 155 | Caffeine metabolism | 0.005 | 0.015 | 0.001 |

PA, primary aldosteronism; FDR: false discovery rate.

**Supplementary Table 5: Metabolic pathways with statistical difference between PA patients and primary hypertension patients.**

| **No.** | **Metabolic pathways** | **p value** | **FDR-adjusted p value** | **Fold Change (PA/Primary hypertension)** |
| --- | --- | --- | --- | --- |
| 1 | Glycosphingolipid biosynthesis - lacto and neolacto series | 0.017 | 0.031 | 3.351 |
| 2 | Phospholipase D signaling pathway | 0.004 | 0.010 | 1.665 |
| 3 | Axon regeneration | 0.004 | 0.011 | 1.647 |
| 4 | Choline metabolism in cancer | 0.004 | 0.010 | 1.633 |
| 5 | Autophagy - yeast | 0.001 | 0.003 | 1.578 |
| 6 | Phenylpropanoid biosynthesis | 0.002 | 0.006 | 1.429 |
| 7 | Other glycan degradation | 0.026 | 0.047 | 1.324 |
| 8 | Prolactin signaling pathway | 0.007 | 0.015 | 1.303 |
| 9 | Sphingolipid metabolism | 0.012 | 0.024 | 1.281 |
| 10 | NOD-like receptor signaling pathway | 0.001 | 0.003 | 1.279 |
| 11 | Cyanoamino acid metabolism | 0.002 | 0.006 | 1.273 |
| 12 | Neomycin, kanamycin and gentamicin biosynthesis | 0.007 | 0.015 | 1.261 |
| 13 | AMPK signaling pathway | < 0.001 | 0.001 | 1.258 |
| 14 | Prostate cancer | < 0.001 | 0.001 | 1.256 |
| 15 | Antigen processing and presentation | < 0.001 | 0.001 | 1.256 |
| 16 | Progesterone-mediated oocyte maturation | < 0.001 | 0.001 | 1.256 |
| 17 | Th17 cell differentiation | < 0.001 | 0.001 | 1.256 |
| 18 | IL-17 signaling pathway | < 0.001 | 0.001 | 1.256 |
| 19 | Estrogen signaling pathway | < 0.001 | 0.001 | 1.256 |
| 20 | Epithelial cell signaling in Helicobacter pylori infection | < 0.001 | 0.002 | 1.252 |
| 21 | Glycosphingolipid biosynthesis - globo and isoglobo series | 0.013 | 0.025 | 1.249 |
| 22 | Biosynthesis of ansamycins | 0.001 | 0.002 | 1.242 |
| 23 | Plant-pathogen interaction | 0.001 | 0.003 | 1.235 |
| 24 | Galactose metabolism | 0.001 | 0.005 | 1.234 |
| 25 | Histidine metabolism | < 0.001 | 0.001 | 1.222 |
| 26 | Glucosinolate biosynthesis | < 0.001 | < 0.001 | 1.220 |
| 27 | Secondary bile acid biosynthesis | 0.001 | 0.002 | 1.218 |
| 28 | Biosynthesis of vancomycin group antibiotics | 0.002 | 0.006 | 1.205 |
| 29 | beta-Lactam resistance | < 0.001 | 0.002 | 1.200 |
| 30 | Polyketide sugar unit biosynthesis | 0.004 | 0.010 | 1.198 |
| 31 | PI3K-Akt signaling pathway | 0.001 | 0.002 | 1.196 |
| 32 | Necroptosis | < 0.001 | 0.001 | 1.192 |
| 33 | Valine, leucine and isoleucine biosynthesis | < 0.001 | 0.001 | 1.191 |
| 34 | Insulin resistance | < 0.001 | 0.001 | 1.190 |
| 35 | 2-Oxocarboxylic acid metabolism | < 0.001 | 0.001 | 1.187 |
| 36 | Carbapenem biosynthesis | < 0.001 | 0.001 | 1.186 |
| 37 | Salmonella infection | 0.001 | 0.004 | 1.181 |
| 38 | Starch and sucrose metabolism | 0.001 | 0.003 | 1.181 |
| 39 | Photosynthesis | < 0.001 | 0.001 | 1.173 |
| 40 | Vancomycin resistance | < 0.001 | 0.001 | 1.170 |
| 41 | Acarbose and validamycin biosynthesis | 0.005 | 0.012 | 1.168 |
| 42 | Streptomycin biosynthesis | 0.009 | 0.019 | 1.166 |
| 43 | Biosynthesis of amino acids | < 0.001 | 0.001 | 1.166 |
| 44 | MicroRNAs in cancer | 0.015 | 0.029 | 1.163 |
| 45 | Biofilm formation - Escherichia coli | 0.005 | 0.012 | 1.161 |
| 46 | Biosynthesis of various secondary metabolites - part 2 | 0.002 | 0.006 | 1.159 |
| 47 | Phenylalanine, tyrosine and tryptophan biosynthesis | 0.001 | 0.004 | 1.158 |
| 48 | Amino sugar and nucleotide sugar metabolism | 0.004 | 0.011 | 1.157 |
| 49 | Monobactam biosynthesis | 0.002 | 0.005 | 1.156 |
| 50 | Zeatin biosynthesis | 0.006 | 0.014 | 1.154 |
| 51 | Novobiocin biosynthesis | 0.002 | 0.006 | 1.153 |
| 52 | RNA degradation | < 0.001 | 0.002 | 1.150 |
| 53 | Pentose and glucuronate interconversions | 0.008 | 0.018 | 1.150 |
| 54 | Glutamatergic synapse | 0.014 | 0.027 | 1.148 |
| 55 | Pyrimidine metabolism | < 0.001 | 0.001 | 1.143 |
| 56 | Legionellosis | 0.013 | 0.025 | 1.142 |
| 57 | Peptidoglycan biosynthesis | < 0.001 | 0.001 | 1.141 |
| 58 | Alanine, aspartate and glutamate metabolism | 0.001 | 0.005 | 1.140 |
| 59 | Homologous recombination | < 0.001 | 0.001 | 1.138 |
| 60 | C5-Branched dibasic acid metabolism | 0.002 | 0.006 | 1.135 |
| 61 | Biotin metabolism | 0.002 | 0.006 | 1.135 |
| 62 | Lysine biosynthesis | 0.001 | 0.002 | 1.130 |
| 63 | Porphyrin and chlorophyll metabolism | 0.023 | 0.041 | 1.129 |
| 64 | Pantothenate and CoA biosynthesis | < 0.001 | 0.002 | 1.128 |
| 65 | One carbon pool by folate | 0.007 | 0.015 | 1.127 |
| 66 | Ribosome | < 0.001 | 0.001 | 1.127 |
| 67 | Proteoglycans in cancer | 0.001 | 0.003 | 1.127 |
| 68 | RNA transport | 0.001 | 0.002 | 1.127 |
| 69 | Thiamine metabolism | 0.003 | 0.008 | 1.126 |
| 70 | Phosphatidylinositol signaling system | 0.015 | 0.029 | 1.125 |
| 71 | Insulin signaling pathway | 0.023 | 0.041 | 1.125 |
| 72 | Selenocompound metabolism | 0.005 | 0.012 | 1.120 |
| 73 | Bacterial secretion system | 0.006 | 0.014 | 1.119 |
| 74 | Aminoacyl-tRNA biosynthesis | 0.001 | 0.002 | 1.116 |
| 75 | Tropane, piperidine and pyridine alkaloid biosynthesis | 0.015 | 0.029 | 1.114 |
| 76 | Arginine biosynthesis | 0.004 | 0.011 | 1.114 |
| 77 | Cysteine and methionine metabolism | 0.001 | 0.004 | 1.113 |
| 78 | Biosynthesis of secondary metabolites | 0.001 | 0.004 | 1.113 |
| 79 | Nitrogen metabolism | 0.001 | 0.005 | 1.113 |
| 80 | Oxidative phosphorylation | 0.002 | 0.005 | 1.112 |
| 81 | Antifolate resistance | 0.014 | 0.027 | 1.112 |
| 82 | Sulfur relay system | 0.009 | 0.019 | 1.111 |
| 83 | Drug metabolism - other enzymes | 0.004 | 0.010 | 1.108 |
| 84 | Nucleotide excision repair | 0.001 | 0.003 | 1.107 |
| 85 | Mismatch repair | 0.001 | 0.004 | 1.107 |
| 86 | Fluid shear stress and atherosclerosis | 0.004 | 0.010 | 1.106 |
| 87 | HIF-1 signaling pathway | 0.006 | 0.014 | 1.103 |
| 88 | Carbon fixation pathways in prokaryotes | 0.010 | 0.021 | 1.101 |
| 89 | DNA replication | 0.002 | 0.006 | 1.101 |
| 90 | Glucagon signaling pathway | 0.004 | 0.011 | 1.100 |
| 91 | Cell cycle - Caulobacter | 0.002 | 0.006 | 1.099 |
| 92 | Glycerophospholipid metabolism | 0.006 | 0.013 | 1.097 |
| 93 | Purine metabolism | 0.006 | 0.014 | 1.097 |
| 94 | Carbon fixation in photosynthetic organisms | 0.002 | 0.007 | 1.094 |
| 95 | Protein export | 0.011 | 0.022 | 1.093 |
| 96 | Pentose phosphate pathway | 0.014 | 0.027 | 1.091 |
| 97 | Nicotinate and nicotinamide metabolism | 0.003 | 0.009 | 1.087 |
| 98 | D-Glutamine and D-glutamate metabolism | 0.008 | 0.016 | 1.087 |
| 99 | Human papillomavirus infection | < 0.001 | 0.002 | 1.086 |
| 100 | Viral carcinogenesis | < 0.001 | 0.002 | 1.086 |
| 101 | Ribosome biogenesis in eukaryotes | 0.001 | 0.003 | 1.083 |
| 102 | Prodigiosin biosynthesis | 0.007 | 0.015 | 1.080 |
| 103 | Glycine, serine and threonine metabolism | 0.011 | 0.022 | 1.079 |
| 104 | Fatty acid biosynthesis | 0.008 | 0.018 | 1.077 |
| 105 | Glycolysis / Gluconeogenesis | 0.018 | 0.033 | 1.077 |
| 106 | Base excision repair | 0.005 | 0.012 | 1.073 |
| 107 | Carbon metabolism | 0.007 | 0.015 | 1.072 |
| 108 | Methane metabolism | 0.023 | 0.041 | 1.070 |
| 109 | Terpenoid backbone biosynthesis | 0.015 | 0.029 | 1.069 |
| 110 | Alzheimer disease | 0.026 | 0.047 | 0.887 |
| 111 | Inositol phosphate metabolism | 0.015 | 0.029 | 0.855 |
| 112 | MAPK signaling pathway - plant | 0.012 | 0.024 | 0.822 |
| 113 | Valine, leucine and isoleucine degradation | 0.016 | 0.031 | 0.821 |
| 114 | Apoptosis - fly | 0.005 | 0.012 | 0.817 |
| 115 | Flavonoid biosynthesis | 0.006 | 0.013 | 0.815 |
| 116 | Stilbenoid, diarylheptanoid and gingerol biosynthesis | 0.006 | 0.013 | 0.815 |
| 117 | Glutathione metabolism | 0.001 | 0.004 | 0.794 |
| 118 | Lysine degradation | 0.004 | 0.010 | 0.775 |
| 119 | Pathways in cancer | 0.002 | 0.005 | 0.746 |
| 120 | Ubiquinone and other terpenoid-quinone biosynthesis | 0.005 | 0.012 | 0.743 |
| 121 | African trypanosomiasis | 0.024 | 0.044 | 0.687 |
| 122 | Arachidonic acid metabolism | 0.001 | 0.003 | 0.671 |
| 123 | Benzoate degradation | 0.001 | 0.002 | 0.666 |
| 124 | Arabinogalactan biosynthesis - Mycobacterium | 0.013 | 0.025 | 0.655 |
| 125 | Tryptophan metabolism | 0.004 | 0.010 | 0.655 |
| 126 | Dioxin degradation | 0.007 | 0.015 | 0.650 |
| 127 | Huntington disease | 0.002 | 0.006 | 0.638 |
| 128 | Amyotrophic lateral sclerosis (ALS) | 0.002 | 0.006 | 0.619 |
| 129 | Chlorocyclohexane and chlorobenzene degradation | 0.016 | 0.031 | 0.608 |
| 130 | Aminobenzoate degradation | < 0.001 | 0.001 | 0.562 |
| 131 | Platinum drug resistance | < 0.001 | 0.001 | 0.539 |
| 132 | Metabolism of xenobiotics by cytochrome P450 | 0.003 | 0.008 | 0.532 |
| 133 | Thyroid hormone synthesis | < 0.001 | < 0.001 | 0.530 |
| 134 | Linoleic acid metabolism | 0.006 | 0.013 | 0.526 |
| 135 | Drug metabolism - cytochrome P450 | 0.002 | 0.006 | 0.521 |
| 136 | Xylene degradation | 0.001 | 0.004 | 0.507 |
| 137 | Atrazine degradation | 0.003 | 0.008 | 0.499 |
| 138 | Staphylococcus aureus infection | 0.002 | 0.005 | 0.472 |
| 139 | Synthesis and degradation of ketone bodies | < 0.001 | < 0.001 | 0.458 |
| 140 | alpha-Linolenic acid metabolism | 0.002 | 0.006 | 0.444 |
| 141 | RIG-I-like receptor signaling pathway | 0.003 | 0.008 | 0.403 |
| 142 | Bladder cancer | 0.004 | 0.010 | 0.401 |
| 143 | Ethylbenzene degradation | 0.001 | 0.003 | 0.375 |
| 144 | Biosynthesis of unsaturated fatty acids | 0.001 | 0.004 | 0.370 |
| 145 | Betalain biosynthesis | 0.001 | 0.003 | 0.338 |
| 146 | Cushing syndrome | < 0.001 | < 0.001 | 0.334 |
| 147 | Renal cell carcinoma | < 0.001 | < 0.001 | 0.334 |
| 148 | Renin-angiotensin system | < 0.001 | < 0.001 | 0.329 |
| 149 | Mineral absorption | < 0.001 | 0.002 | 0.329 |
| 150 | Non-homologous end-joining | 0.016 | 0.031 | 0.322 |
| 151 | Styrene degradation | < 0.001 | < 0.001 | 0.308 |
| 152 | Geraniol degradation | < 0.001 | 0.001 | 0.273 |
| 153 | Toluene degradation | < 0.001 | < 0.001 | 0.200 |
| 154 | Hepatocellular carcinoma | < 0.001 | < 0.001 | 0.198 |
| 155 | Caprolactam degradation | < 0.001 | 0.001 | 0.193 |
| 156 | Chemical carcinogenesis | < 0.001 | < 0.001 | 0.180 |
| 157 | Fluorobenzoate degradation | < 0.001 | < 0.001 | 0.166 |
| 158 | Meiosis - yeast | 0.001 | 0.002 | 0.126 |
| 159 | Cardiac muscle contraction | < 0.001 | < 0.001 | 0.117 |
| 160 | Lipoarabinomannan (LAM) biosynthesis | < 0.001 | < 0.001 | 0.085 |
| 161 | Carotenoid biosynthesis | < 0.001 | < 0.001 | 0.041 |
| 162 | Retrograde endocannabinoid signaling | 0.005 | 0.011 | 0.037 |
| 163 | Cocaine addiction | < 0.001 | < 0.001 | 0.032 |
| 164 | Serotonergic synapse | < 0.001 | < 0.001 | 0.032 |
| 165 | Alcoholism | < 0.001 | < 0.001 | 0.032 |
| 166 | Amphetamine addiction | < 0.001 | < 0.001 | 0.032 |
| 167 | Dopaminergic synapse | < 0.001 | < 0.001 | 0.032 |
| 168 | Furfural degradation | 0.003 | 0.007 | 0.030 |
| 169 | Parkinson disease | < 0.001 | < 0.001 | 0.026 |
| 170 | Polycyclic aromatic hydrocarbon degradation | < 0.001 | < 0.001 | 0.020 |
| 171 | Herpes simplex virus 1 infection | < 0.001 | < 0.001 | 0.020 |
| 172 | Non-alcoholic fatty liver disease (NAFLD) | < 0.001 | < 0.001 | 0.020 |
| 173 | Hepatitis B | < 0.001 | < 0.001 | 0.019 |
| 174 | Measles | < 0.001 | < 0.001 | 0.019 |
| 175 | Apoptosis - multiple species | < 0.001 | < 0.001 | 0.019 |
| 176 | Colorectal cancer | < 0.001 | < 0.001 | 0.019 |
| 177 | Epstein-Barr virus infection | < 0.001 | < 0.001 | 0.019 |
| 178 | Viral myocarditis | < 0.001 | < 0.001 | 0.019 |
| 179 | p53 signaling pathway | < 0.001 | < 0.001 | 0.019 |
| 180 | Hepatitis C | < 0.001 | < 0.001 | 0.019 |
| 181 | Small cell lung cancer | < 0.001 | < 0.001 | 0.019 |
| 182 | Influenza A | < 0.001 | < 0.001 | 0.019 |
| 183 | Human immunodeficiency virus 1 infection | < 0.001 | < 0.001 | 0.019 |
| 184 | Human cytomegalovirus infection | < 0.001 | < 0.001 | 0.019 |
| 185 | Kaposi sarcoma-associated herpesvirus infection | < 0.001 | < 0.001 | 0.019 |
| 186 | Toxoplasmosis | < 0.001 | < 0.001 | 0.017 |
| 187 | Vibrio cholerae infection | 0.001 | 0.003 | 0.015 |
| 188 | Basal transcription factors | < 0.001 | < 0.001 | 0.012 |
| 189 | Photosynthesis - antenna proteins | < 0.001 | < 0.001 | 0.010 |
| 190 | Sesquiterpenoid and triterpenoid biosynthesis | < 0.001 | < 0.001 | 0.007 |
| 191 | Steroid biosynthesis | < 0.001 | < 0.001 | 0.007 |
| 192 | Staurosporine biosynthesis | 0.002 | 0.006 | 0.004 |
| 193 | Steroid degradation | < 0.001 | < 0.001 | 0.002 |
| 194 | Sphingolipid signaling pathway | < 0.001 | < 0.001 | 0.002 |
| 195 | Bisphenol degradation | < 0.001 | < 0.001 | 0.002 |
| 196 | Monoterpenoid biosynthesis | 0.012 | 0.025 | 0.002 |
| 197 | Renin secretion | 0.002 | 0.005 | 0.002 |
| 198 | Isoflavonoid biosynthesis | 0.003 | 0.008 | 0.001 |
| 199 | Cholinergic synapse | 0.003 | 0.008 | 0.001 |
| 200 | Indole alkaloid biosynthesis | 0.012 | 0.025 | 0.001 |
| 201 | Systemic lupus erythematosus | 0.002 | 0.005 | 0.001 |
| 202 | Melanogenesis | 0.001 | 0.002 | 0.001 |
| 203 | Parathyroid hormone synthesis, secretion and action | < 0.001 | < 0.001 | < 0.001 |
| 204 | Caffeine metabolism | < 0.001 | < 0.001 | < 0.001 |

PA, primary aldosteronism; FDR: false discovery rate.

**Supplementary Table 6: Metabolic pathways with statistical difference between primary hypertension patients and healthy controls.**

| **No.** | **Metabolic pathways** | **P value** | **FDR-adjusted P value** | **Fold Change (Primary hypertension/Control)** |
| --- | --- | --- | --- | --- |
| 1 | Cholinergic synapse | 0.013 | 0.048 | 4.930 |
| 2 | Parathyroid hormone synthesis, secretion and action | < 0.001 | 0.007 | 3.878 |
| 3 | Caffeine metabolism | 0.002 | 0.022 | 2.870 |
| 4 | Bisphenol degradation | 0.002 | 0.022 | 2.831 |
| 5 | Stilbenoid, diarylheptanoid and gingerol biosynthesis | 0.003 | 0.023 | 2.790 |
| 6 | Flavonoid biosynthesis | 0.003 | 0.023 | 2.790 |
| 7 | Plant hormone signal transduction | 0.013 | 0.048 | 2.715 |
| 8 | Photosynthesis - antenna proteins | 0.004 | 0.024 | 2.471 |
| 9 | Steroid biosynthesis | 0.001 | 0.014 | 2.433 |
| 10 | Sesquiterpenoid and triterpenoid biosynthesis | 0.002 | 0.021 | 2.212 |
| 11 | Staphylococcus aureus infection | < 0.001 | 0.007 | 2.206 |
| 12 | Carotenoid biosynthesis | 0.002 | 0.022 | 2.189 |
| 13 | Serotonergic synapse | 0.004 | 0.023 | 2.072 |
| 14 | Dopaminergic synapse | 0.004 | 0.023 | 2.071 |
| 15 | Cocaine addiction | 0.004 | 0.023 | 2.069 |
| 16 | Alcoholism | 0.004 | 0.023 | 2.068 |
| 17 | Amphetamine addiction | 0.004 | 0.023 | 2.068 |
| 18 | Fluorobenzoate degradation | 0.001 | 0.020 | 1.997 |
| 19 | Sphingolipid signaling pathway | 0.015 | 0.048 | 1.990 |
| 20 | Toluene degradation | 0.001 | 0.013 | 1.983 |
| 21 | D-Arginine and D-ornithine metabolism | 0.007 | 0.033 | 1.913 |
| 22 | Steroid degradation | 0.009 | 0.039 | 1.888 |
| 23 | Caprolactam degradation | 0.001 | 0.021 | 1.865 |
| 24 | Bladder cancer | 0.006 | 0.029 | 1.833 |
| 25 | Polycyclic aromatic hydrocarbon degradation | 0.004 | 0.024 | 1.833 |
| 26 | Chemical carcinogenesis | 0.002 | 0.021 | 1.821 |
| 27 | Biosynthesis of unsaturated fatty acids | 0.002 | 0.022 | 1.814 |
| 28 | Renal cell carcinoma | 0.001 | 0.013 | 1.802 |
| 29 | Cushing syndrome | 0.001 | 0.013 | 1.802 |
| 30 | Geraniol degradation | 0.001 | 0.021 | 1.792 |
| 31 | Carbohydrate digestion and absorption | < 0.001 | 0.007 | 1.782 |
| 32 | RIG-I-like receptor signaling pathway | 0.003 | 0.023 | 1.777 |
| 33 | Basal transcription factors | 0.012 | 0.046 | 1.743 |
| 34 | Lipoarabinomannan (LAM) biosynthesis | 0.007 | 0.033 | 1.742 |
| 35 | Hepatocellular carcinoma | 0.003 | 0.023 | 1.720 |
| 36 | Ethylbenzene degradation | 0.004 | 0.023 | 1.714 |
| 37 | Arabinogalactan biosynthesis - Mycobacterium | < 0.001 | 0.007 | 1.708 |
| 38 | Ubiquinone and other terpenoid-quinone biosynthesis | < 0.001 | 0.007 | 1.684 |
| 39 | Mineral absorption | 0.007 | 0.033 | 1.681 |
| 40 | Styrene degradation | 0.004 | 0.024 | 1.666 |
| 41 | Betalain biosynthesis | 0.012 | 0.046 | 1.659 |
| 42 | alpha-Linolenic acid metabolism | 0.006 | 0.029 | 1.658 |
| 43 | African trypanosomiasis | 0.006 | 0.029 | 1.646 |
| 44 | Chagas disease (American trypanosomiasis) | 0.005 | 0.028 | 1.629 |
| 45 | Lipopolysaccharide biosynthesis | < 0.001 | 0.007 | 1.628 |
| 46 | Linoleic acid metabolism | 0.007 | 0.032 | 1.611 |
| 47 | Cardiac muscle contraction | 0.015 | 0.048 | 1.611 |
| 48 | Xylene degradation | 0.003 | 0.023 | 1.572 |
| 49 | Drug metabolism - cytochrome P450 | 0.002 | 0.022 | 1.568 |
| 50 | Biosynthesis of siderophore group nonribosomal peptides | 0.004 | 0.024 | 1.560 |
| 51 | Atrazine degradation | 0.013 | 0.048 | 1.557 |
| 52 | Metabolism of xenobiotics by cytochrome P450 | 0.002 | 0.022 | 1.549 |
| 53 | Penicillin and cephalosporin biosynthesis | < 0.001 | 0.007 | 1.495 |
| 54 | Toxoplasmosis | 0.015 | 0.048 | 1.456 |
| 55 | Lipoic acid metabolism | < 0.001 | 0.007 | 1.454 |
| 56 | Chlorocyclohexane and chlorobenzene degradation | 0.015 | 0.048 | 1.451 |
| 57 | Thyroid hormone synthesis | 0.001 | 0.013 | 1.450 |
| 58 | Phosphotransferase system (PTS) | < 0.001 | 0.007 | 1.447 |
| 59 | Retinol metabolism | 0.004 | 0.023 | 1.424 |
| 60 | Dioxin degradation | 0.011 | 0.043 | 1.421 |
| 61 | Aminobenzoate degradation | 0.003 | 0.023 | 1.408 |
| 62 | Pancreatic secretion | 0.008 | 0.035 | 1.404 |
| 63 | MAPK signaling pathway - fly | 0.006 | 0.029 | 1.396 |
| 64 | Longevity regulating pathway | 0.008 | 0.035 | 1.383 |
| 65 | Herpes simplex virus 1 infection | 0.015 | 0.048 | 1.379 |
| 66 | Kaposi sarcoma-associated herpesvirus infection | 0.015 | 0.048 | 1.376 |
| 67 | Human cytomegalovirus infection | 0.015 | 0.048 | 1.376 |
| 68 | Human immunodeficiency virus 1 infection | 0.015 | 0.048 | 1.375 |
| 69 | Small cell lung cancer | 0.015 | 0.048 | 1.375 |
| 70 | Viral myocarditis | 0.015 | 0.048 | 1.374 |
| 71 | Colorectal cancer | 0.015 | 0.048 | 1.374 |
| 72 | Epstein-Barr virus infection | 0.015 | 0.048 | 1.374 |
| 73 | Apoptosis - multiple species | 0.015 | 0.048 | 1.374 |
| 74 | p53 signaling pathway | 0.015 | 0.048 | 1.374 |
| 75 | Influenza A | 0.016 | 0.048 | 1.374 |
| 76 | Measles | 0.015 | 0.048 | 1.374 |
| 77 | Hepatitis B | 0.015 | 0.048 | 1.374 |
| 78 | Hepatitis C | 0.016 | 0.048 | 1.373 |
| 79 | Synthesis and degradation of ketone bodies | 0.008 | 0.035 | 1.360 |
| 80 | Apoptosis - fly | 0.001 | 0.013 | 1.340 |
| 81 | Huntington disease | 0.004 | 0.025 | 1.338 |
| 82 | MAPK signaling pathway - plant | < 0.001 | 0.009 | 1.328 |
| 83 | Glutathione metabolism | < 0.001 | 0.007 | 1.325 |
| 84 | Proximal tubule bicarbonate reclamation | 0.004 | 0.025 | 1.320 |
| 85 | Platinum drug resistance | 0.011 | 0.043 | 1.319 |
| 86 | Tryptophan metabolism | 0.004 | 0.023 | 1.318 |
| 87 | Valine, leucine and isoleucine degradation | 0.001 | 0.013 | 1.302 |
| 88 | Degradation of aromatic compounds | 0.003 | 0.023 | 1.294 |
| 89 | Amyotrophic lateral sclerosis (ALS) | 0.012 | 0.046 | 1.277 |
| 90 | Arachidonic acid metabolism | 0.008 | 0.035 | 1.270 |
| 91 | Lysine degradation | 0.001 | 0.013 | 1.269 |
| 92 | Benzoate degradation | 0.004 | 0.023 | 1.257 |
| 93 | Phosphonate and phosphinate metabolism | 0.001 | 0.021 | 1.220 |
| 94 | Thermogenesis | 0.004 | 0.024 | 1.214 |
| 95 | Ascorbate and aldarate metabolism | 0.012 | 0.046 | 1.184 |
| 96 | Isoquinoline alkaloid biosynthesis | 0.009 | 0.038 | 1.181 |
| 97 | Cationic antimicrobial peptide (CAMP) resistance | 0.002 | 0.022 | 1.168 |
| 98 | Tyrosine metabolism | 0.002 | 0.021 | 1.167 |
| 99 | Pathways in cancer | 0.004 | 0.024 | 1.167 |
| 100 | Phenylalanine metabolism | 0.002 | 0.022 | 1.166 |
| 101 | Fatty acid degradation | 0.005 | 0.027 | 1.159 |
| 102 | Inositol phosphate metabolism | 0.011 | 0.043 | 1.156 |
| 103 | Biofilm formation - Vibrio cholerae | 0.001 | 0.013 | 1.154 |
| 104 | beta-Alanine metabolism | 0.004 | 0.024 | 1.147 |
| 105 | Citrate cycle (TCA cycle) | 0.001 | 0.013 | 1.142 |
| 106 | Longevity regulating pathway - multiple species | 0.016 | 0.048 | 1.128 |
| 107 | Arginine and proline metabolism | 0.002 | 0.022 | 1.115 |
| 108 | Central carbon metabolism in cancer | 0.008 | 0.035 | 1.104 |
| 109 | Folate biosynthesis | 0.003 | 0.023 | 1.101 |
| 110 | Propanoate metabolism | 0.008 | 0.035 | 1.090 |
| 111 | Glyoxylate and dicarboxylate metabolism | 0.012 | 0.046 | 1.088 |
| 112 | Butanoate metabolism | 0.004 | 0.024 | 1.086 |
| 113 | D-Alanine metabolism | 0.001 | 0.019 | 1.082 |
| 114 | Pathogenic Escherichia coli infection | 0.008 | 0.035 | 1.069 |
| 115 | Riboflavin metabolism | 0.004 | 0.024 | 1.062 |
| 116 | Glucosinolate biosynthesis | 0.015 | 0.048 | 0.929 |
| 117 | Necroptosis | 0.012 | 0.046 | 0.914 |
| 118 | Insulin resistance | 0.005 | 0.027 | 0.909 |
| 119 | Biosynthesis of various secondary metabolites - part 2 | 0.003 | 0.023 | 0.887 |
| 120 | NOD-like receptor signaling pathway | 0.012 | 0.046 | 0.880 |
| 121 | PI3K-Akt signaling pathway | 0.007 | 0.033 | 0.875 |
| 122 | Biosynthesis of ansamycins | 0.007 | 0.034 | 0.862 |
| 123 | AMPK signaling pathway | 0.001 | 0.013 | 0.827 |
| 124 | Flagellar assembly | 0.011 | 0.043 | 0.816 |
| 125 | Bacterial chemotaxis | 0.006 | 0.029 | 0.812 |
| 126 | Glycosphingolipid biosynthesis - lacto and neolacto series | 0.014 | 0.048 | 0.307 |

FDR: false discovery rate.


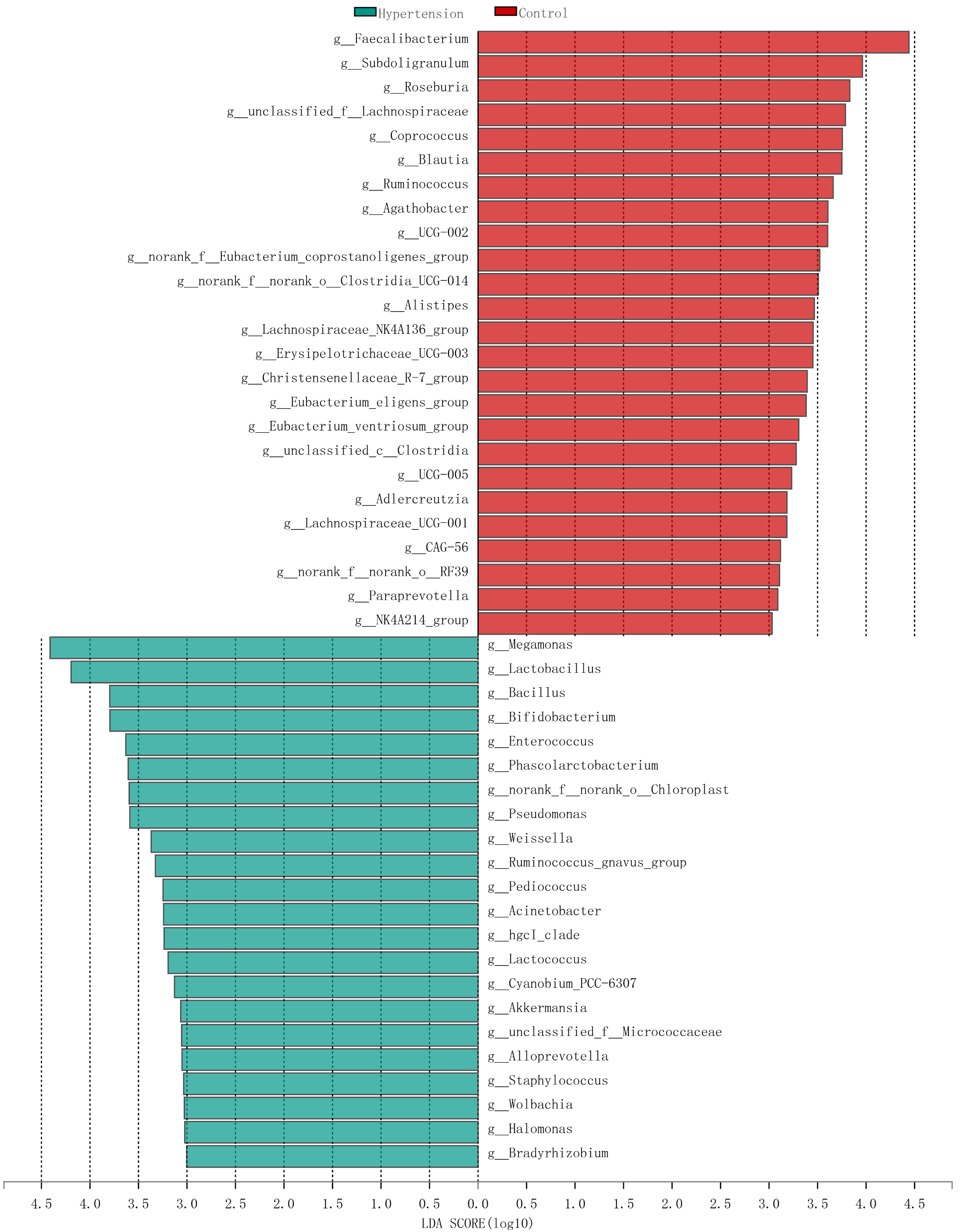


**Supplementary Figure 1. Different intestinal bacteria between primary hypertension patients and healthy controls.** Red bars are genera with higher relative abundances in healthy controls. Green bars are genera with higher relative abundances in primary hypertension patients.


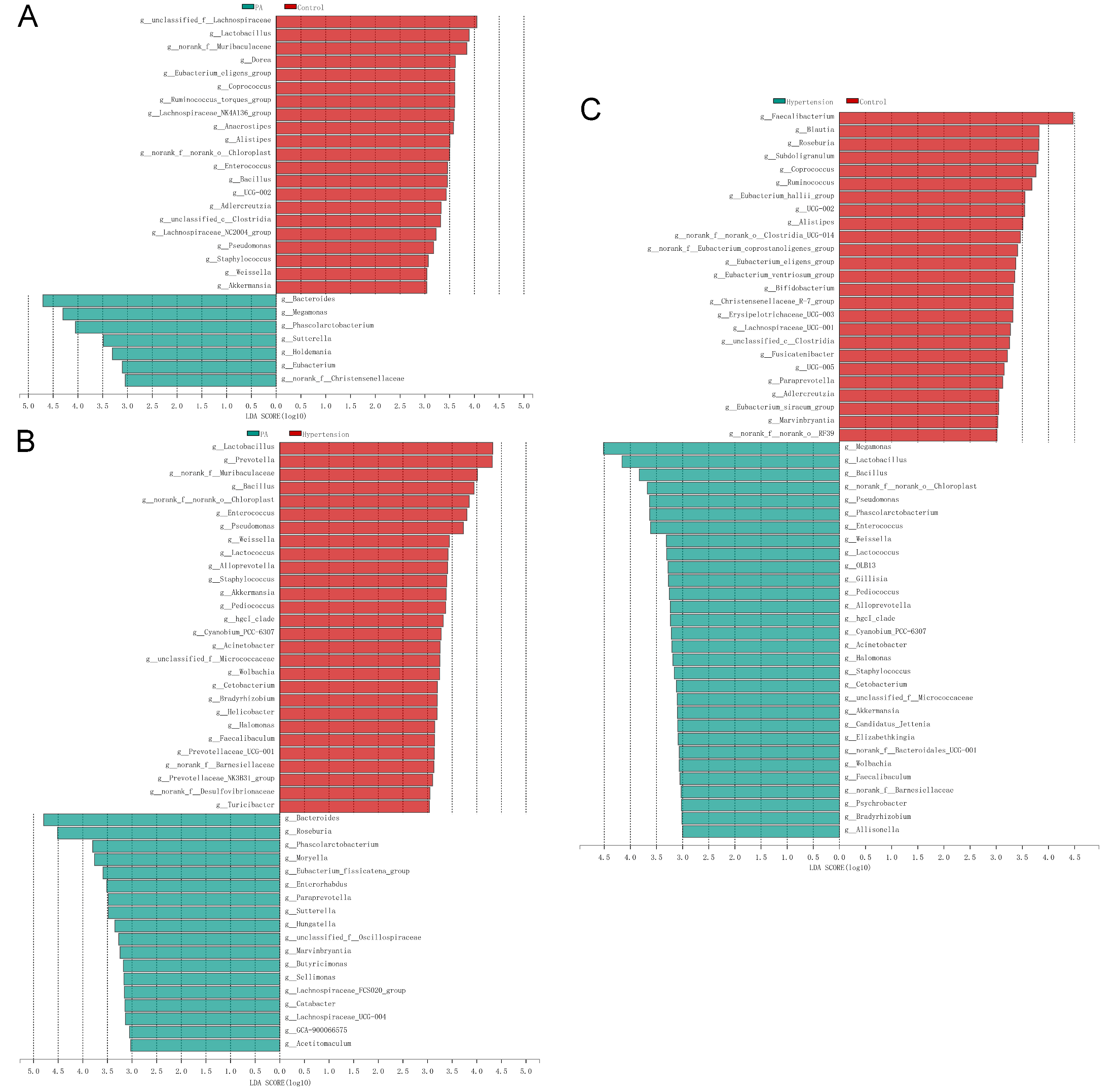


**Supplementary Figure 2. Differences in intestinal bacteria between PA patients, primary hypertension patients and healthy controls without diabetes mellitus.** (A) PA patients vs. healthy controls. Red bars are genera with higher relative abundances in healthy controls. Green bars are genera with higher relative abundances in PA patients. (B) PA patients vs. primary hypertension patients. Red bars are genera with higher relative abundances in primary hypertension patients. Green bars are genera with higher relative abundances in PA patients. (C) Primary hypertension patients vs. healthy controls. Red bars are genera with higher relative abundances in healthy controls. Green bars are genera with higher relative abundances in primary hypertension patients.
